# Acute Pancreatitis Related to COVID-19 Infection: A Systematic Review and Analysis of Data

**DOI:** 10.7759/cureus.28380

**Published:** 2022-08-25

**Authors:** Ahmed Ali Aziz, Muhammad Ali Aziz, Maleeha Saleem, Muhammad Haseeb ul Rasool

**Affiliations:** 1 Internal Medicine, Saint Francis Medical Center, Trenton, USA; 2 Internal Medicine, BronxCare Health System, New York City, USA; 3 Medicine, Icahn School of Medicine at Mount Sinai, Queens Hospital Center, New York City, USA

**Keywords:** adverse outcomes of sars-cov-2, necrotising pancreatitis, acute pancreatitis due to covid-19, covid 19, acute pancreatitis

## Abstract

There is increasing literature mentioning severe acute respiratory syndrome coronavirus 2 (SARS-CoV-2) infection (COVID-19 infection) causing acute pancreatitis (AP). It is hypothesized that SARS-Cov-2 causes pancreatic injury either by direct cytotoxic effect of the virus on pancreatic cells through the angiotensin-converting enzyme 2 (ACE2) receptors - the main receptors for the virus located on pancreatic cells - or by the cytokine storm that results from COVID-19 infection or a component of both. Many viruses are related to AP including mumps, coxsackievirus, cytomegalovirus (CMV), Epstein-Barr virus (EBV), and as data evolves SARS-CoV-2 virus may be one of them as well. We conducted a systematic literature review to explore the current literature and provide an overview of the evidence of AP in COVID-19 infection. We studied the presence of AP in patients with SARS-CoV-2 infection and calculated the time of diagnosis of SARS-CoV-2 infection with respect to the time of diagnosis of AP. We also studied the age, gender, clinical manifestations, time of onset of symptoms, laboratory values, imaging findings, mortality, length of stay, comorbidities, need for Intensive Care Unit (ICU) care, and excluded any other common causes of AP. We included 40 articles comprising 46 patients. All patients had a positive SARS-CoV-2 polymerase chain reaction (PCR) test and all patients had AP as per Atlanta’s criteria. The most common clinical presentation was abdominal pain in 29 (63.0%). Edematous pancreas was the most common Computed Tomography Abdomen Pelvis (CTAP) scan finding in these patients (35 patients). Seventeen (37%) patients required ICU admission and six (13%) patients died. Our study provides an important overview of the available data on AP in COVID-19 patients and concludes that AP is an important complication in COVID-19 infection and should be considered as an important differential in patients with COVID-19 infection who complain of abdominal pain.

## Introduction and background

The most common clinical manifestations of severe acute respiratory syndrome coronavirus 2 (SARS-CoV-2) infection (COVID-19 infection) are respiratory, particularly shortness of breath, cough, and sore throat [1]. But, as the number of cases of SARS-CoV-2 has increased across the world, other symptoms and clinical scenarios have emerged. Among these are gastrointestinal (GI) and hepatic involvement. The GI involvement includes gastroparesis, gastritis, enteritis, colitis, and pancreatitis. GI involvement has been hypothesized to be mediated by the expression of angiotensin-converting enzyme 2 (ACE2) receptors on the GI tract which are the main receptors of SARS-CoV-2 [2, 3].

The pancreas also expresses ACE2 receptors at levels greater than expressed in the lungs. This renders the pancreas a potential target for SARS-CoV-2 [4]. SARS-CoV-2 RNA has been identified in the GI tract including the pancreas by numerous authors [2, 5-7] and many case reports have described acute pancreatitis (AP) as the initial manifestation in patients with COVID-19 infection even before respiratory symptoms [8-11]. There is still uncertainty about the pathophysiological mechanisms involved and the precise etiology of pancreatic injury in the reported cases, however, there are two potential mechanisms hypothesized of how the pancreas is involved in COVID-19 infection: the direct cytotoxic effect of the virus or a delayed injury by the immune response [12-14]. We conducted a literature review to explore the relationship between SARS-CoV-2 infection and AP. The aim of this systematic review was to determine the clinical, imaging, and laboratory features in adult patients with COVID-19 infection and AP and to determine if there is any relationship between AP and COVID-19 infection.

## Review

Methods

A systematic review was conducted according to PRISMA guidelines [15]. We searched PubMed (MEDLINE), Cochrane Library, and clinicaltrials.gov databases till January 3rd, 2022 for published articles using medical subject headings keywords “COVID-19” OR “SARS-CoV-2” and “acute pancreatitis”. Any study (case report or case series) was considered eligible if it contained the original data on at least one SARS-CoV-2-infected individual diagnosed with AP. Only human studies were eligible. We included pregnant patient population. We excluded pediatric patient population. We did not include letters to editors. Studies with COVID-19 infection and elevated lipase or amylase levels but without an established diagnosis of AP according to the revised Atlanta’s criteria were excluded from our systematic review. The literature search was restricted to articles in the English language only. Articles from all across the world were included if they were written in the English language or if an English translation was available. Only full-text articles were included in this review. References of eligible manuscripts were screened for additional articles. Citations were exported to a reference management program (Microsoft Excel 2020, Microsoft® Corp., Redmond, WA).

**Figure 1:**
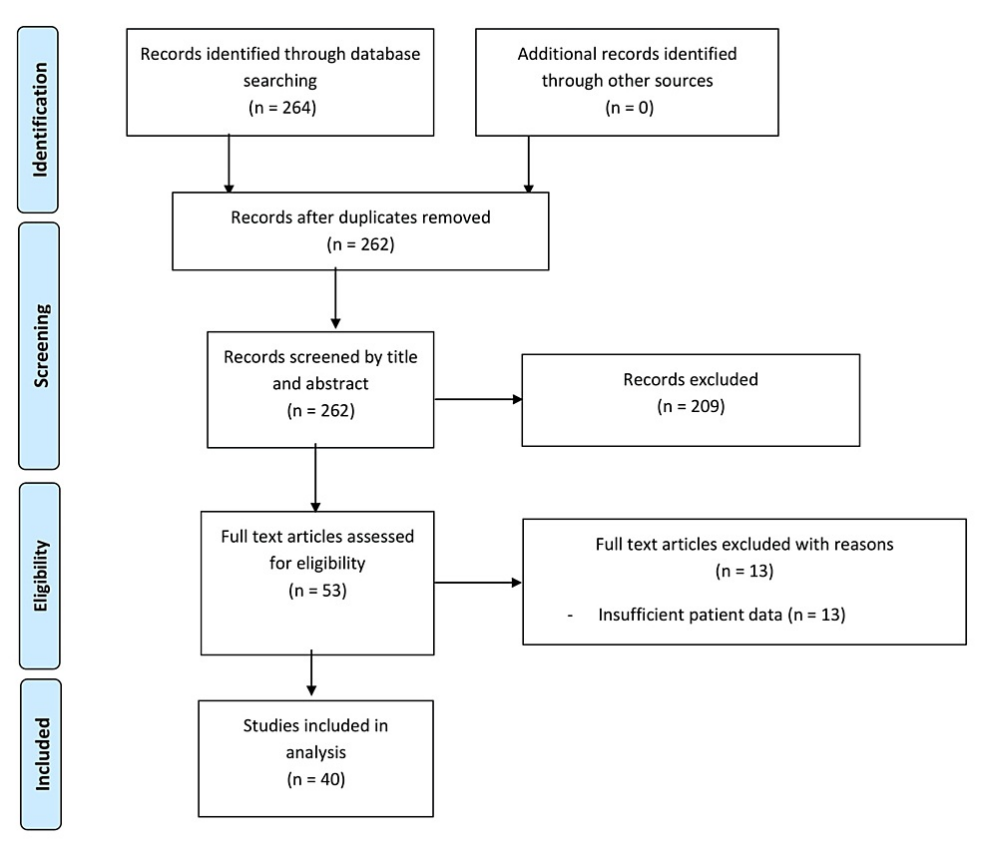
PRISMA flow diagram demonstrating the selection of studies to be included in the review.

The titles and abstracts of studies retrieved by the search strategy and those from additional sources, namely by cross-referencing, were screened independently by two authors (Aziz A and Saleem M) to identify studies that met the predefined inclusion criteria. The initial search strategy yielded 264 articles. After removing duplicates 262 articles were left. A total of 123 articles were excluded on the basis of title as they did not meet the inclusion criteria of our study. Eighty-six articles were excluded on abstract read and 13 articles were excluded on full-text read (Figure 1). Hence, 40 articles were included in the systematic review. These were 35 case reports and five case series. Any disagreements over the eligibility of any study were resolved through discussion and opinion from other authors and a consensus was met in all included papers.

Data were extracted from each of the papers undergoing full-text review and included the author names, year of publication, study design, population number, age, gender, whether SARS-CoV-2 PCR was positive prior to admission (PTA), on admission (OA) or after admission (AA), clinical manifestation on admission, lipase level, amylase level, aspartate transaminase (AST) level, alanine transaminase (ALT) level, Computed Tomography Abdomen Pelvis (CTAP) scan findings, comorbidities, length of stay (LOS), need for ICU care and mortality if any. Descriptive statistics were used to analyze the data.

Results

The final collection included 40 articles; 35 case reports and five case series. This resulted in a total patient population of 46 patients. The average age was 48 years. The youngest patient was 24 years old and the oldest patient was 87 years old (range 63 years). Twenty-six (56.5%) patients were females and 19 (42.2%) patients were males. The gender of one patient was not mentioned. One patient was pregnant. All patients had positive SARS-CoV-2 polymerase chain reaction (PCR) test. All patients met the diagnosis of AP based on the revised Atlanta’s criteria requiring two of (1) abdominal pain, (2) elevated serum amylase or lipase > 3 the upper normal limit, and (3) characteristic findings on diagnostic imaging of edematous pancreatitis or necrotizing pancreatitis [16].

Ten (26.7%) patients had positive SARS-CoV-2 PCR test PTA. This was usually done at an outside facility because patients were either having symptoms of COVID-19 infection like dyspnea, cough, fever, and myalgia or had exposure to a confirmed COVID-19 positive patient. On average, these patients had a positive SARS-CoV-2 PCR test seven days prior to admission. Out of these 10 patients, nine patients met Atlanta’s criteria for AP on admission/presentation to the hospital while one patient developed AP later during hospitalization.

Twenty-six (56.5%) patients had a positive SARS-CoV-2 PCR test on the day of presentation to the hospital. The SARS-CoV-2 PCR test was done either because patients had symptoms of COVID-19 (dyspnea, cough, fever, and myalgia), or unexplained bilateral infiltrates on chest radiograph or as a routine SARS-CoV-2 PCR test done prior to hospital admission. Sixteen (61.5%) of these patients met Atlanta’s criteria for AP at the same time as they were positive for COVID-19. Ten (38.5%) patients developed AP later during the hospitalization course.

Ten (26.7%) patients had positive SARS-CoV-2 PCR test after admission to the hospital. These patients were initially admitted to the hospital for AP treatment. They later developed dyspnea or had ground glass opacities in bilateral lower lungs incidentally detected on CT scan abdomen pelvis. For these reasons, a SARS-CoV-2 PCR test was done on these patients which turned out to be positive.

Overall, the most common clinical presentation was abdominal pain in 29 (63.0%) patients followed by fever and dyspnea each in 21 (45.7%) patients (Table 1).

**Table 1:** Clinical presentation on admission

Clinical Presentation	Number of Patients (Percentage)
Abdominal Pain	29 (63.0%)
Fever	21 (45.7%)
Dyspnea	21 (45.7%)
Nausea / Vomiting	16 (34.8%)
Cough	11 (23.9%)
Sore throat	5 (10.9%)
Malaise	6 (13%)
Headache	3 (6.5%)

Serum lipase and amylase were elevated in 34 out of 37 patients (92%) and 31 of 34 (91%) patients, where the data were available, respectively. Twenty-three out of 37 patients (62%) and 22 of 34 (64%) patients had serum lipase and amylase x3 upper limits respectively. The mean lipase level was 1390, amylase was 885, aspartate transaminase (AST) was 53 and alanine transaminase (ALT) was 59.

Thirty-five (76.1%) patients had findings of edematous pancreas and three (6.5%) patients had findings of necrotizing pancreas on CTAP scan. Two (4.3%) patients had normal pancreas on CTAP scan. CTAP scan was not done in one (2.2%) patient due to pregnancy and CTAP scan details were not mentioned in five (10.9%) patients (Tables 2-4).

**Table 2:** CTAP (Computed Tomography Abdomen Pelvis) scan findings

CTAP Scan Findings	Number of Patients (Percentage)
Results not mentioned	5 (10.9%) patients
Not done	1 (2.2%) patient
Edematous Pancreas	35 (76.1%) patients
Necrotizing Pancreas	3 (6.5%) patients
Normal Pancreas	2 (4.3%) patients

**Table 3:** Summary of case reports on acute pancreatitis and COVID-19 included in the review. ?: Not mentioned in article; CR: Case Report; M: Male; F: Female; N/V: Nausea / Vomiting; PTA: Prior to admission; OA: On admission; AA: After admission; (P): Pregnant; AP: Acute Pancreatitis; CTAP: Computed Tomography Abdomen Pelvis; LOS: Length of Stay.

No.	Reference	Year	Study Design	Population No.	Gender	Age	Symptoms OA	COVID-19 diagnosis PTA, OA, or AA	AP OA or AA	CTAP Scan Findings of Pancreas	Lipase	Amylase	ICU Stay	LOS	Mortality
1	Aloysius et al. [9]	2020	CR	1	F	36	Abdominal Pain, Fever, Dyspnea, N/V, Cough	OA	OA	Normal	627	325	Yes	?	No
2	Meireles et al. [17]	?	CR	1	F	36	Fever, Dyspnea, Cough	OA	AA	Normal	631	718	No	?	No
3	Mohammadi Arbati and Molseghi [18]	2021	CR	1	M	28	Abdominal Pain, Dyspnea	OA	OA	Edematous	1273	758	Yes	15	No
4	Yamamoto et al. [19]	2021	CR	1	F	70	Fever, Sore throat	OA	AA	Edematous	381	238	No	?	No
5	Lakshmanan and Malik [20]	2020	CR	1	M	68	N/V	PTA	AA	Edematous	2035	1030	No	?	No
6	AlHarmi et al. [21]	2021	CR	1	F	52	Fever, Dyspnea, Cough	OA	AA	Edematous	?	?	No	23	No
7	Purayil et al. [22]	2020	CR	1	M	58	Fever, N/V	OA	OA	?	600	249	No	?	No
8	Bokhari and Mahmood [23]	2020	CR	1	M	32	Fever, Cough, Sore throat, Malaise	OA	AA	Edematous	721	672	No	10	No
9	Kripalani et al. [24]	2021	CR	1	F	79	Abdominal Pain, Fever, Sore throat	AA	OA	Necrotizing	6178	1075	Yes	?	No
10	Tollard et al. [25]	2021	CR	1	F	32	Fever, Dyspnea	AA	AA	Edematous	321	?	Yes	78	Yes
11	Sandhu et al. [26]	2021	CR	1	F	25	Abdominal Pain, Fever, Dyspnea	OA	OA	Edematous	35	?	Yes	2	Yes
12	Basukala et al. [27]	2021	CR	1	F	49	Abdominal Pain, Fever, Dyspnea, Cough	OA	OA	Edematous	568	1563	Yes	?	Yes
13	Chandra et al. [28]	2021	CR	1	M	53	Abdominal Pain, Dyspnea	OA	OA	Edematous	1200	?	Yes	30	No
14	Patnaik et al. [29]	2020	CR	1	M	29	Abdominal Pain, Fever, Dyspnea	OA	OA	Edematous	1650	2861	No	?	No
15	Brikman et al. [30]	2020	CR	1	M	61	Fever, Dyspnea, Cough	OA	AA	Edematous	203	142	No	?	No
16	Wifi et al. [31]	2021	CR	1	F	72	Abdominal Pain	PTA	OA	?	710	1667	Yes	?	No
17	da Costa Ferreira et al. [32]	2021	CR	1	M	35	Abdominal Pain	AA	OA	Edematous	?	1609	Yes	12	No
18	Mazrouei et al. [33]	2020	CR	1	M	24	Abdominal Pain	PTA	OA	Edematous	578	391	No	?	No
19	Kataria et al. [34]	2020	CR	1	F	49	Dyspnea	AA	AA	Edematous	1541	501	No	?	No
20	Kandasamy [35]	2020	CR	1	F	45	Abdominal Pain	AA	OA	Edematous	293	364	No	?	No
21	Mansour et al. [36]	2021	CR	1	M	47	Abdominal Pain	PTA	OA	Edematous	?	2708	No	?	No
22	Simou et al. [37]	2020	CR	1	?	67	Fever, Dyspnea, Malaise	OA	AA	Edematous	576	?	Yes	18	Yes
23	Cheung et al. [38]	2020	CR	1	M	38	Abdominal Pain, Fever	PTA	OA	Edematous	?	?	No	?	No
24	Ghosh et al. [39]	2020	CR	1	M	69	Fever, Dyspnea	OA	AA	Edematous	412	58	No	?	No
25	Kumaran et al. [40]	2020	CR	1	F	67	Abdominal Pain, N/V	OA	OA	Necrotizing	?	1483	No	?	No
26	Hatch-Vallier et al. [41]	2021	CR	1	F	39	Dyspnea, N/V	OA	OA	Edematous	43	?	No	?	No
27	Alves et al. [42]	2020	CR	1	F	56	Abdominal Pain, Dyspnea, Cough, Malaise	AA	AA	Edematous	583	544	Yes	35	No
28	Gadiparthi et al. [43]	2021	CR	1	F	74	Abdominal Pain, Dyspnea, Cough	OA	OA	Edematous	7500	229	Yes	35	No
29	Rabice et al. [44]	2020	CR	1	F	36 (P)	Fever, Malaise	OA	AA	Not done	875	88	No	?	No
30	Muhammad Abrar Jeelani et al. [45]	202	CR	1	M	24	Abdominal Pain, N/V	PTA	OA	Edematous	2025	?	No	?	No
31	Eldaly et al. [46]	2021	CR	1	M	44	Abdominal Pain, N/V	PTA	OA	Edematous	256	773	No	?	No
32	Alwaeli et al. [47]	2020	CR	1	M	30	Dyspnea, Fever, Malaise	OA	OA	Edematous	1022	151	No	?	No
33	Al Armashi et al. [48]	2021	CR	1	M	37	Abdominal Pain	OA	OA	Edematous	5418	264	Yes	?	No
34	Sudarsanam et al. [49]	2021	CR	1	M	35	Abdominal Pain, Fever, Cough	AA	OA	Necrotizing	40	40	No	5	No
35	Gupta et al. [50]	2021	CR	1	F	25	Fever, Cough, Sore Throat, Headache	AA	AA	Edematous	2052	1814	No	16	No

**Table 4:** Summary of case series on acute pancreatitis and COVID-19 included in the review. ?: Not mentioned in article; CS: Case Series; M: Male; F: Female; N/V: Nausea / Vomiting; PTA: Prior to admission; OA: On admission; AA: After admission; AP: Acute Pancreatitis; CTAP: Computed Tomography Abdomen Pelvis; LOS: Length of Stay.

No.	Reference	Year	Study Design	Population No.	Gender	Age	Symptoms OA	COVID-19 diagnosis PTA, OA or AA	AP OA or AA	CTAP Scan Findings of Pancreas	Lipase	Amylase	ICU Stay	LOS	Mortality
1	Hadi et al. [6]	2020	CS	2	F	47	Fever, Dyspnea, Sore throat, Headache	OA	OA	?	?	1500	Yes	?	No
					F	68	Abdominal Pain, Fever, N/V, Malaise	OA	AA	?	?	934	Yes	?	No
2	Wang et al. [51]	2020	CS	2	M	42	Abdominal Pain, Dyspnea, N/V	AA	OA	Edematous	382	131	Yes	8	Yes
					M	35	Abdominal Pain, N/V	AA	OA	Edematous	1042	?	No	15	No
3	Schembri Higgans et al. [52]	2021	CS	3	F	63	Abdominal Pain, N/V	PTA	OA	?	?	1079	No	?	No
					F	87	Abdominal Pain, N/V	PTA	OA	Edematous	?	499	No	5	No
					F	64	Abdominal Pain, N/V	OA	OA	Edematous	?	2141	No	14	No
4	Berrichi et al. [53]	2021	CS	2	F	36	Dyspnea, Cough, Headache	OA	AA	Edematous	2570	?	Yes	22	Yes
						51	Abdominal Pain, Dyspnea, N/V	OA	OA	Edematous	676	?	No	4	No
5	Amé and Balderramo [54]	2021	CS	2	F	42	Abdominal Pain, N/V	OA	OA	Edematous	2799	2263	No	8	No
					F	65	Abdominal Pain, N/V	PTA	OA	Edematous	1950	470	No	?	No

None of the patients had any of the co-conditions that could precipitate AP like gall stones, hypertriglyceridemia, or alcohol intoxication. If in any article authors had any suspicion for any other entity causing AP it was excluded in full-text review. Hypertension and diabetes mellitus type 2 were the most common comorbidities whenever present. Fourteen (30.4%) patients reported no associated comorbidities. Seventeen (37%) patients required ICU stay. Out of these six (13%) died. The average length of hospital stay was 18 days.

The included studies and their findings are summarized in Table 3 and Table 4.

Discussion

In our systematic review, we summarized available reports of patients with AP having COVID-19 infection. COVID-19 is a multisystem disease with various clinical manifestations ranging from mild upper respiratory symptoms to acute respiratory distress syndrome (ARDS) to the involvement of the GI tract. GI involvement is likely mediated by the expression of ACE2 receptors on the GI tract which are the main receptors of SARS-CoV-2 [1, 2]. The pancreas also expresses ACE2 receptors and hence is a potential target for SARS-CoV-2 [55]. Pancreatic injury in COVID-19 infection might occur by the direct cytotoxic effect of the virus through the ACE2 receptors on the pancreatic cells or by the COVID-19 infection-induced cytokine storm causing multiorgan dysfunction, including pancreatic injury [13, 14]. The evidence for direct pancreatic injury is the detection of SARS CoV-2 RNA in a pancreatic pseudocyst sample by Schepis et al. [56].

As per our review, COVID-19-associated AP occurs in each sex and at every age, in patients with and without comorbidities. Most studies did not mention ethnicity hence, we could not determine whether COVID-19-associated AP is more in certain ethnicities. AP related to COVID-19 can occur in pregnant patients. Our review showed that COVID-19-related AP was more common in females. AP in COVID-19 may be diagnosed and reported at the same time as the diagnosis of SARS-CoV-2 or after several days of the initial diagnosis. There are no set number of days when symptoms of AP manifest after COVID-19 infection. As per our review, it is also possible that patients may only have symptoms of AP and be incidentally positive for COVID-19 infection. The most common symptom on the presentation for patients with COVID-19-related AP was abdominal pain followed by dyspnea and fever. The course of AP in COVID-19 patients is most frequently not severe. Prognosis is commonly determined by pneumonia in COVID-19 patients. Of the 17 patients who required ICU stay all were secondary to ARDS requiring either intubation or high levels of non-invasive ventilation. Of the six patients who died all had ARDS likely secondary to COVID infection of the lungs. Treatment of AP in COVID-19 infection is typical for AP in general and includes intravenous fluids, analgesics, anti-emetics, encouraging the early resumption of diet, and antiviral treatment for COVID-19 infection. Our review does show that SARS-CoV-2 can be a new potential etiological infectious factor of AP. All reported cases had AP confirmed using the revised Atlanta’s criteria. All patients had a positive PCR test for confirmation of SARS-CoV-2 infection. Therefore, our review provides strong evidence for an association between AP and COVID-19.

However, despite the fact that our review indicates that SARS-CoV-2 may cause AP, the following must be taken into account. The abdominal pain in patients with COVID-19 may be due to SARS-CoV-2 injury to the GI tract rather than AP, and it may not be possible to differentiate them. Also, SARS-CoV-2 causing serum lipase and/or amylase elevation is not diagnostic for AP because serum lipase elevation is not specific for pancreatic pathology, and can be seen in other GI pathology, including gastroparesis, gastritis, enteritis, and colitis [57] which are also recognized to be part of the COVID-19 clinical picture. Hence, it cannot definitely be concluded that the abdominal pain and lipase and/or amylase elevations in these patients were secondary to AP and not some other GI pathology blurring the relationship between COVID-19 infection causing AP.

Also, the causal relationship of AP related to COVID-19 infection is challenging, because other potential etiological factors must be ruled out which were not completely ruled out in the articles included in our review. This includes the drugs/antivirals used in the treatment of COVID-19 disease. Some patients in our literature review developed AP after admission to the hospital for SARS-CoV-2 infection. It could be that the treatment for COVID-19 infection like steroids, remdesivir, antibiotics or other pharmacologic drugs had precipitated AP in these patients. In some cases, AP may not be a consequence of SARS-CoV-2 infection but may coexist independently from COVID-19.

Limitations of our study

The quality and consistency of the reports was a major limitation of this review. In some reports, the timing of SARS-CoV-2 diagnosis was not mentioned in exact days when diagnosed prior to admission or after admission. In some reports, the exact time in days of AP diagnosis was not mentioned when diagnosed after admission. The reports claimed that other common causes of AP had been excluded by patients' history, lab work, and imaging, however, an overlap with less frequent etiologies like autoimmune, and medications could not be excluded in at least some of the reports. Values for lipase, amylase and length of stay were missing from a few reports. For these reasons, the quality of case reports is a major limiting factor in our study.

## Conclusions

Our systematic review summarizes AP as a potential complication of COVID-19 infection. All 46 patients had confirmed SARS-CoV-2 infection and all patients developed AP at some time during the course of their COVID-19 infection. Sixteen (34.7%) patients had a diagnosis of AP and SARS-CoV-2 at the same time. Twenty (43.5%) patients which is almost half the population had SARS-CoV-2 diagnosed first and later developed AP. The most common clinical presentation was abdominal pain. Only three (6.5%) patients had findings of necrotizing pancreatitis. The most common causes of AP including gall stones, hypertriglyceridemia, and alcoholism were excluded from all patients. Most of the patients recovered, almost 17 (37%) required critical care stay and six (13%) died. Our knowledge of the exact causal relationship between AP and COVID-19 infection is limited and needs to be supplemented by further large multicenter cohort studies involving large numbers of patients. Our review is important in the sense that it provides an overview of current evidence on AP in COVID-19 patients and tells that AP should be considered as an important differential in a SARS-CoV-2 positive patient with abdominal pain.
